# Inferring signaling pathways with probabilistic programming

**DOI:** 10.1093/bioinformatics/btaa861

**Published:** 2020-12-29

**Authors:** David Merrell, Anthony Gitter

**Affiliations:** Department of Computer Sciences, University of Wisconsin–Madison, Madison, WI 53706, USA; Morgridge Institute for Research, Madison, WI 53715, USA; Department of Computer Sciences, University of Wisconsin–Madison, Madison, WI 53706, USA; Morgridge Institute for Research, Madison, WI 53715, USA; Department of Biostatistics and Medical Informatics, University of Wisconsin–Madison, Madison, WI 53726, USA

## Abstract

**Motivation:**

Cells regulate themselves via dizzyingly complex biochemical processes called signaling pathways. These are usually depicted as a network, where nodes represent proteins and edges indicate their influence on each other. In order to understand diseases and therapies at the cellular level, it is crucial to have an accurate understanding of the signaling pathways at work. Since signaling pathways can be modified by disease, the ability to infer signaling pathways from condition- or patient-specific data is highly valuable. A variety of techniques exist for inferring signaling pathways. We build on past works that formulate signaling pathway inference as a Dynamic Bayesian Network structure estimation problem on phosphoproteomic time course data. We take a Bayesian approach, using Markov Chain Monte Carlo to estimate a posterior distribution over possible Dynamic Bayesian Network structures. Our primary contributions are (i) a novel proposal distribution that efficiently samples sparse graphs and (ii) the relaxation of common restrictive modeling assumptions.

**Results:**

We implement our method, named Sparse Signaling Pathway Sampling, in Julia using the Gen probabilistic programming language. Probabilistic programming is a powerful methodology for building statistical models. The resulting code is modular, extensible and legible. The Gen language, in particular, allows us to customize our inference procedure for biological graphs and ensure efficient sampling. We evaluate our algorithm on simulated data and the HPN-DREAM pathway reconstruction challenge, comparing our performance against a variety of baseline methods. Our results demonstrate the vast potential for probabilistic programming, and Gen specifically, for biological network inference.

**Availability and implementation:**

Find the full codebase at https://github.com/gitter-lab/ssps.

**Supplementary information:**

Supplementary data are available at *Bioinformatics* online.

## 1 Introduction

Signaling pathways enable cells to process information rapidly in response to external environmental changes or intracellular cues. One of the core signaling mechanisms is protein phosphorylation. Kinases add phosphate groups to substrate proteins and phosphatases remove them. These changes in phosphorylation state can act as switches, controlling proteins’ activity and function. A protein’s phosphorylation status affects its localization, stability and interaction partners (Newman *et al.*, 2014). Ultimately, phosphorylation changes regulate important biological processes, such as transcription and cell growth, death and differentiation (Hunter, 2009; Kholodenko *et al*., 2010).

Pathway databases characterize the signaling relationships among groups of proteins but are not tailored to individual biological contexts. Even for well-studied pathways, such as epidermal growth factor receptor-mediated signaling, the proteins significantly phosphorylated during a biological response can differ greatly from those in the curated pathway (Köksal *et al.*, 2018). The discrepancy can be due to context-specific signaling (Hill *et al.*, 2017), cell type-specific protein abundances or signaling rewiring in disease (Pawson and Warner, 2007). Therefore, there is a need to learn context-specific signaling pathway representations from observed phosphorylation changes. In the clinical setting, patient-specific signaling pathway representations may eventually be able to guide therapeutic decisions (Drake *et al.*, 2016; Eduati *et al.*, 2020; Halasz *et al.*, 2016).

Diverse classes of techniques have been developed to model and infer signaling pathways (Kholodenko *et al.*, 2012). They take approaches including Granger causality (Carlin *et al.*, 2017; Shojaie and Michailidis, 2010), information theory (Cheong *et al.*, 2011; Krishnaswamy *et al.*, 2014), logic models (Eker *et al.*, 2002; Gjerga *et al.*, 2020; Guziolowski *et al.*, 2013), differential equations (Henriques *et al.*, 2017; Molinelli *et al.*, 2013; Schoeberl *et al.*, 2002), non-parametric statistical tests (Zhang and Song, 2013) and probabilistic graphical models (Sachs *et al.*, 2005) among others. Some signaling pathway reconstruction algorithms take advantage of perturbations, such as receptor stimulation or kinase inhibition. Although perturbing individual pathway members can causally link them to downstream phosphorylation changes, characterizing a complex pathway can require a large number of perturbation experiments. Inferring pathway structure from temporal phosphorylation data presents an attractive alternative. A single time series phosphorylation dataset can reveal important dynamics without perturbing individual pathway members. For instance, a kinase cannot phosphorylate substrates before it is activated.

An alternative approach to pathway reconstruction selects a context-specific subnetwork from a general background network. These algorithms can use phosphorylation data to assign scores to protein nodes in a protein–protein interaction network. They then select edges that connect the high-scoring nodes, generating a subnetwork that may explain how the induced phosphorylation changes arise from the source of stimulation. Extensions accommodate temporal scores on the nodes (Budak *et al.*, 2015; Köksal *et al.*, 2018; Norman and Cicek, 2019; Patil *et al.*, 2013).

Our present work builds on past techniques that formulate signaling pathway inference as a Dynamic Bayesian Network (DBN) structure estimation problem. This family of techniques relies on two core ideas: (i) we can use a DBN to model phosphorylation time series data and (ii) the DBN’s structure translates directly to a directed graph representing the signaling pathway. Rather than identifying a single DBN that best fits the data, these techniques take a Bayesian approach—they yield a *posterior distribution* over possible DBN structures. Some techniques use Markov Chain Monte Carlo (MCMC) to sample from the posterior (Gregorczyk, 2010; Werhli and Husmeier, 2007). Others use exact, enumerative inference to compute posterior probabilities (Hill *et al.*, 2012; Oates *et al.*, 2014; Spencer *et al.*, 2015).

We present a new Bayesian DBN-based technique, Sparse Signaling Pathway Sampling (SSPS). It improves on past MCMC methods by using a novel proposal distribution specially tailored for the large, sparse graphs prevalent in biological applications. Furthermore, SSPS makes weaker modeling assumptions than other DBN approaches. As a result, SSPS scales to larger problem sizes and yields superior predictions in comparison to other DBN techniques.

We implement SSPS using the Gen probabilistic programming language (PPL). Probabilistic programming is a powerful methodology for building statistical models. It enables the programmer to build models in a legible, modular, reusable fashion. This flexibility was important for prototyping and developing the current form of SSPS and readily supports future improvements or extensions.

## 2 Materials and methods

### 2.1 Model formulation

SSPS makes specific modeling assumptions. We start with the DBN model of Hill *et al.* (2012), relax some assumptions and modify it in other ways to be better-suited for MCMC inference.

#### 2.1.1 Preliminary definitions

We first define some notation for clarity’s sake. Let *G* denote a *directed graph* with vertices *V* and edges *E*(*G*). Graph *G* will represent a signaling pathway, with vertices *V* corresponding to proteins and edges *E*(*G*) indicating their influence relationships. We use ${\text{pa}}_{G}(i)$ to denote the *parents* of vertex *i* in *G*.

Let *X* denote our time series data, consisting of |V| variables measured at *T* timepoints. *X* is a T×|V| matrix where the *j*th column corresponds to the *j*th variable and the *j*th graph vertex. As a convenient shorthand, let $X_{+}$ denote the *latest* T−1 timepoints in *X*, and let $X_{-}$ denote the *earliest* T−1 timepoints in *X*. Lastly, define $B_{j}\equiv X_{-,{\text{pa}}_{G}(j)}$. In other words, *B_j_* contains the values of variable *j’*s parents at the T−1 earliest timepoints. In general, *B_j_* may also include columns of non-linear interactions between the parents. We will only include linear terms, unless stated otherwise.

#### 2.1.2 Model derivation

In our setting, we aim to infer *G* from *X*. In particular, Bayesian approaches seek a *posterior distribution* P(G|X) over possible graphs. From Bayes’ rule, we know P(G|X)∝P(X|G)·P(G). That is, a Bayesian model is fully specified by its choice of *prior distribution P*(*G*) and *likelihood function* P(X|G).

We derive our model from the one used by Hill *et al.* (2012). They choose a prior distribution of the form:

(1) P(G | G′,λ)∝ exp ⁡(−λ|E(G)∖E(G′)|),

parameterized by a *reference graph* G′ and *inverse temperature λ*. This prior gives uniform probability to all subgraphs of G′ and ‘penalizes’ edges not contained in E(G′). *λ* controls the ‘importance’ given to the reference graph.

Hill *et al.* choose a Gaussian DBN for their likelihood function. Intuitively, they assume linear relationships between variables and their parents:

$$X_{+,j}∼N(B_{j}\beta_{j},\sigma_{j}^{2})\;\forall j\in \{1\ldots |V|\}.$$

A suitable prior over the regression coefficients *β_j_* and noise parameters $\sigma_{j}^{2}$ (Fig. 1) allows us to integrate them out, yielding this *marginal likelihood function*:

(2) $$P(X|G)\propto \prod_{j=1}^{\left|V\right|}T^{-\frac{\left|{\text{pa}}_{G}(j)\right|}{2}}{\left(X_{+,j}^{⊤}X_{+,j}-\frac{T-1}{T}X_{+,j}^{⊤}(B_{j}{\hat{\beta }}_{\mathrm{ols}})\right)}^{-\frac{T-1}{2}},$$

where ${\hat{\beta }}_{\mathrm{ols}}={\left(B_{j}^{⊤}B_{j}\right)}^{-1}B_{j}^{⊤}X_{+,j}$ is the ordinary least squares estimate of *β_j_*. For notational simplicity, Equation (2) assumes we have a single time course of length *T*. In general, there may be multiple time course replicates with differing lengths. The marginal likelihood generalizes to that case in a straightforward way.

**Figure:**
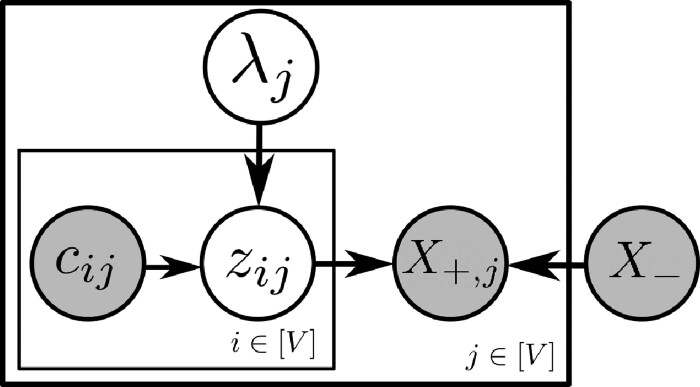
$\lambda_{j}∼\text{Uniform}\;(\lambda_{\text{min}},\lambda_{\text{max}})\;\forall j\in \{1\ldots |V|\}$ $z_{ij}\;|\;c_{ij},\lambda_{j}∼\text{Bernoulli}\left(\frac{e^{-\lambda_{j}}}{e^{-c_{ij}\lambda_{j}}+e^{-\lambda_{j}}}\right)\;\forall i,j\in \{1\ldots |V|\}$ $\sigma_{j}^{2}\propto \frac{1}{\sigma_{j}^{2}}\;\forall j\in \{1\ldots |V|\}$ $\beta_{j}\;|\;\sigma_{j}^{2}∼N\left(0,T\sigma_{j}^{2}{\left(B_{j}^{⊤}B_{j}\right)}^{-1}\right)\;\forall j\in \{1\ldots |V|\}$ $X_{+,j}\;|\;B_{j},\beta_{j},\sigma_{j}^{2}∼N\left(B_{j}\beta_{j},\sigma_{j}^{2}I\right)\;\forall j\in \{1\ldots |V|\}$ **Fig. 1.** Our generative model. (Top) Plate notation. DBN parameters *β_j_* and $\sigma_{j}^{2}$ have been marginalized out. (Bottom) Full probabilistic specification. We usually set $\lambda_{\text{min}}≃3$ and $\lambda_{\text{max}}=15$. If $\lambda_{\text{min}}>0$ is too small, Markov chains will occasionally be initialized with very large numbers of edges, causing computational issues. The method is insensitive to $\lambda_{\text{max}}$ as long as it is sufficiently large. Notice the improper prior $1/\sigma_{j}^{2}$. In this specification, *B_j_* denotes $X_{-,{\text{pa}}_{Z}(j)}$; i.e. the parents of vertex *j* depend on edge existence variables *Z*

In SSPS, we use the same marginal likelihood function [Equation (2)], but a different prior distribution *P*(*G*). We obtain our prior distribution by decomposing Equation (1) into a product of independent Bernoulli trials over graph edges. This decomposition in turn allows us to make some useful generalizations. Define *edge existence variables* $z_{ij}\equiv \;1[(i,j)\in E(G)]$. Let *Z* be the |V|×|V| matrix of all *z_ij_*. Then, we can rewrite Equation (1) as follows:

$$P(G|G\prime ,\lambda )\;\equiv \;P(Z|G\prime ,\lambda )\;\propto \;\prod_{\left(i,j)\notin E(G\prime \right)}e^{-z_{ij}\lambda }$$

 

$$=\prod_{\left(i,j)\in E(G\prime \right)}{\left(\frac{1}{2}\right)}^{z_{ij}}{\left(\frac{1}{2}\right)}^{1-z_{ij}}\prod_{\left(i,j)\notin E(G\prime \right)}{\left(\frac{e^{-\lambda }}{1+e^{-\lambda }}\right)}^{z_{ij}}{\left(\frac{1}{1+e^{-\lambda }}\right)}^{1-z_{ij}},$$

where the last line is a true equality—it gives a normalized probability measure. We see that the original prior is simply a product of Bernoulli variables parameterized by a shared inverse temperature, *λ*. See Appendix A for a more detailed derivation.

Rewriting the prior in this form opens the door to generalizations. First, we address a shortcoming in the way reference graph G′ expresses prior knowledge. The original prior assigns equal probability to every edge of G′. However, in practice, we may have differing levels of prior confidence in the edges. We address this by allowing a real-valued prior confidence *c_ij_* for each edge:

(3) $$P(Z|C,\lambda )=\prod_{\left(i,j\right)}{\left(\frac{e^{-\lambda }}{e^{-c_{ij}\lambda }+e^{-\lambda }}\right)}^{z_{ij}}{\left(\frac{e^{-c_{ij}\lambda }}{e^{-c_{ij}\lambda }+e^{-\lambda }}\right)}^{1-z_{ij}},$$

where *C* is the matrix of all prior confidences *c_ij_*, replacing G′. Notice that if every $c_{ij}\in \{0,1\}$, then Equation (3) is equivalent to the original prior. In effect, Equation (3)  *interpolates* the original prior, permitting a continuum of confidences on the interval [0,1].

We make one additional change to the prior by replacing the shared *λ* inverse temperature variable with a collection of variables, $\Lambda =\{\lambda_{j}\;|\;j=1,\ldots ,|V|\}$, one for each vertex of the graph. Recall that the original *λ* variable determined the importance of the reference graph. In the new formulation, each *λ_j_* controls the importance of the prior knowledge for vertex *j* and its parents:

(4) $$P(Z|C,\Lambda )=\prod_{\left(i,j\right)}{\left(\frac{e^{-\lambda_{j}}}{e^{-c_{ij}\lambda_{j}}+e^{-\lambda_{j}}}\right)}^{z_{ij}}{\left(\frac{e^{-c_{ij}\lambda_{j}}}{e^{-c_{ij}\lambda_{j}}+e^{-\lambda_{j}}}\right)}^{1-z_{ij}}.$$

We introduced Λ primarily to help MCMC converge more efficiently. Experiments with the shared *λ* revealed a multimodal posterior that tended to trap *λ* in high or low values. The introduction of vertex-specific *λ_j_* variables yielded faster convergence with weaker modeling assumptions—an improvement in both respects.

We implicitly relax the model assumptions further via our inference procedure. For sake of tractability, the original exact method of Hill *et al.* (2012) imposes a hard constraint on the in-degree of each vertex. In contrast, we use a MCMC inference strategy with no in-degree constraints.

In summary, our model departs from that of Hill *et al.* (2012) in three important respects. It permits real-valued prior confidences *C*, introduces vertex-specific inverse temperature variables Λ and places no constraints on vertices’ in-degrees. See the full model in Figure 1 and Appendix A for additional details.

### 2.2 Inference procedure

Our method uses MCMC to infer posterior edge existence probabilities. As described in Section 2.1, our model contains two classes of unobserved random variables: (i) the edge existence variables *Z* and (ii) the inverse temperature variables Λ. For each step of MCMC, we loop through these variables and update them in a Metropolis–Hastings fashion.

#### 2.2.1 Main loop

At a high level, our MCMC procedure consists of a loop over the graph vertices, *V*. For each vertex *j*, we update its inverse temperature variable *λ_j_* and then update its *parent set* ${\text{pa}}_{G}(j)$. All of these updates are Metropolis–Hastings steps; the proposal distributions are described below. Each completion of this loop yields one iteration of the Markov chain.

#### 2.2.2 Proposal distributions

For the inverse temperature variables, we use a symmetric Gaussian proposal: $\lambda_{j}^{\prime }∼N(\lambda_{j},\xi^{2})$. In practice, the method is insensitive to *ξ*; we typically set ξ=3.

The parent set proposal distribution is more complicated. There are two principles at work when we design a graph proposal distribution: (i) the proposal should efficiently traverse the space of directed graphs and (ii) it should favor graphs with higher posterior probability. The most widely used graph proposal distribution selects a *neighboring* graph uniformly from the set of possible ‘add-edge,’ ‘remove-edge’ and ‘reverse-edge’ actions (Gregorczyk, 2010; Werhli and Husmeier, 2007). We will refer to this traditional proposal distribution as the *uniform graph proposal*. In our setting, we expect sparse graphs to be much more probable than dense ones—notice how the marginal likelihood function [Equation (2)] strongly penalizes $\left|{\text{pa}}_{G}(j)\right|$. However, the uniform graph proposal exhibits a preference toward *dense graphs*. It proposes ‘add-edge’ actions too often. This motivates us to design a new proposal distribution tailored for sparse graphs—one that operates on our sparse *parent set* graph representation.

For a given graph vertex j∈V, the parent set proposal distribution updates ${\text{pa}}_{G}(j)$ by choosing from the following actions:

- add-parent. Select one of vertex *j’*s non-parents uniformly at random, and add it to ${\text{pa}}_{G}(j)$.
- remove-parent. Select one of vertex *j’*s parents uniformly at random, and remove it from ${\text{pa}}_{G}(j)$.
- swap-parent. A simultaneous application of add-parent and remove-parent. Perhaps surprisingly, this action is not made redundant by the other two. It plays an important role by yielding updates that maintain the size of the parent set. Because the marginal likelihood [Equation (2)] changes steeply with $\left|{\text{pa}}_{G}(j)\right|$, Metropolis–Hastings acceptance probabilities will be higher for actions that keep $\left|{\text{pa}}_{G}(j)\right|$ constant.

These three actions are sufficient to explore the space of directed graphs, but we need another mechanism to bias the exploration toward *sparse* graphs. We introduce this preference via the *probability* assigned to each action. Intuitively, we craft the action probabilities so that when $\left|{\text{pa}}_{G}(j)\right|$ is too small, add-parent moves are most probable. When $\left|{\text{pa}}_{G}(j)\right|$ is too big, remove-parent moves are most probable. When $\left|{\text{pa}}_{G}(j)\right|$ is about right, all moves are equally probable.

**Fig. 2.**
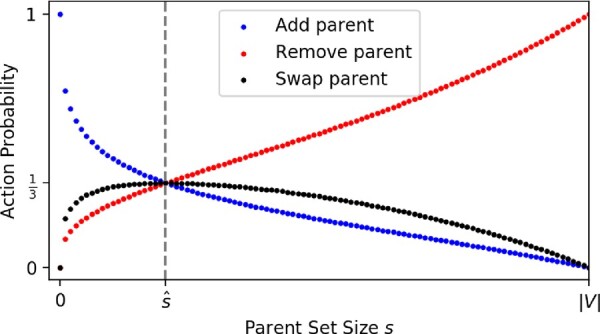
Action probabilities as a function of parent set size. The reference size $\hat{s}$ is determined from prior knowledge. It approximates the size of a ‘typical’ parent set. When $s<\hat{s}$, add-parent is most probable; when $s>\hat{s}$, remove-parent is most probable; and when $s=\hat{s}$, all actions have equal probability

We formulate the action probabilities for vertex *j* as follows. As a shorthand, let $s_{j}=|{\text{pa}}_{G}(j)|$ and define the *reference size* ${\hat{s}}_{j}=\sum_{i=1}^{\left|V\right|}c_{ij}$. That is, ${\hat{s}}_{j}$ uses the prior edge confidences *C* to estimate an appropriate reference size for the parent set. Then, the action probabilities are

$$\begin{array}{c} p(\mathrm{add}-\mathrm{parent}|s_{j},{\hat{s}}_{j})\propto 1-{\left(\frac{s_{j}}{\left|V\right|}\right)}^{\gamma ({\hat{s}}_{j})}\\ p(\mathrm{remove}-\mathrm{parent}|s_{j},{\hat{s}}_{j})\propto {\left(\frac{s_{j}}{\left|V\right|}\right)}^{\gamma ({\hat{s}}_{j})}\\ p(\mathrm{swap}-\mathrm{parent}|s_{j},{\hat{s}}_{j})\propto 2{\left(\frac{s_{j}}{\left|V\right|}\right)}^{\gamma ({\hat{s}}_{j})}\cdot \left(1-{\left(\frac{s_{j}}{\left|V\right|}\right)}^{\gamma ({\hat{s}}_{j})}\right) \end{array},$$

where $\gamma ({\hat{s}}_{j})=1/\;{\text{log}}_{2}(|V|/{\hat{s}}_{j})$. We use these functional forms only because they have certain useful properties: (i) when $s_{j}=0$, the probability of add-parent is 1; (ii) when $s_{j}=|V|$, the probability of remove-parent is 1 and (iii) when $s_{j}={\hat{s}}_{j}$, all actions have equal probability (Fig. 2). Beyond that, these probabilities have no particular justification. We provide additional information about the parent set proposal in Appendix B.

Recall that Metropolis–Hastings requires us to compute the reverse transition probability for any proposal we make. This could pose a challenge given our relatively complicated parent set proposal distribution. However, Gen provides a helpful interface for computing reverse probabilities. The user can provide an *involution* function that returns the reverse of a given action. Gen then manages the reverse probabilities without further intervention. This makes it relatively easy to implement Metropolis–Hastings updates with unusual proposal distributions.

#### 2.2.3 Termination, convergence and inference

We follow the basic MCMC protocols described by Gelman *et al.* (2014). This entails running multiple (i.e. 4) Markov chains and discarding the first half of each chain as burnin. In all of our analyses, we terminate each Markov chain when it either (i) reaches a length of 100 000 iterations or (ii) the execution time exceeds 12 h. These termination conditions are arbitrary but emulate a real-world setting where it may be acceptable to let the method run overnight.

Upon termination, we assess convergence with two diagnostics: Potential Scale Reduction Factor (PSRF) and effective number of samples ($N_{\text{eff}}$). PSRF identifies cases where the Markov chains fail to mix or achieve stationarity. $N_{\text{eff}}$ provides a sense of ‘sample size’ for our inferred quantities. It adjusts the number of MCMC samples by accounting for autocorrelation in each chain. For our purposes, we say a quantity has *failed to converge* if its PSRF ≥1.01 or $N_{\text{eff}}<10$. Note that satisfying these criteria does not guarantee convergence. However, failure to satisfy them is a reliable flag for non-convergence.

Assuming a quantity has not failed to converge, we estimate it by simply taking its sample mean from all samples remaining after burnin. In our setting, we are primarily interested in *edge existence* probabilities; i.e. we compute the fraction of samples containing each edge.

### 2.3 Probabilistic programming implementation

We implemented SSPS using the Gen PPL. We briefly describe the probabilistic programming methodology and its advantages in our setting.

#### 2.3.1 Probabilistic programming

Probabilistic programming is a methodology for building statistical models. It is based on the idea that statistical models are *generative processes*—sequences of operations on random variables. In probabilistic programming, we express the generative process as a program written in a PPL. This program is then compiled to produce a log-probability function, which can be used in inference tasks. Probabilistic programming systems typically provide a set of generic inference methods for performing those tasks—e.g. MCMC or Variational Bayes.

Compare this with a more traditional approach, where the user must (i) derive and implement the log-probability function and (ii) implement an inference method that operates on the log-probability function. This process of manual derivation and implementation is error-prone and requires a high degree of expertise from the user. In contrast, probabilistic programming only requires the user to express their model in a PPL. The probabilistic programming system manages other details. Probabilistic programming also tends to promote good software engineering principles, such as abstraction, modularity and legibility. Most PPLs organize code into functions, which can be reused by multiple statistical models.

Several PPLs have emerged in recent years (see Appendix C and Supplementary Table S1). Examples include Stan (Carpenter *et al.*, 2017), Edward2 (Dillon *et al.*, 2017), Pyro (Bingham *et al.*, 2019), PyMC3 (Salvatier *et al.*, 2016) and Gen (Cusumano-Towner *et al.*, 2019). PPLs differ in how they balance *expressive power* and *ease of use*. For example, Stan makes it easy to build hierarchical statistical models with continuous variables but caters poorly to other model classes. At the other extreme, Gen can readily express a large class of models—discrete and continuous variables with complex relationships—but requires the user to design a customized inference procedure.

#### 2.3.2 Implementation in Gen

We chose the Gen PPL (Cusumano-Towner *et al.*, 2019) for its expressive power and customizable inference. While implementing SSPS, the customizability of Gen allowed us to begin with simple prototypes and then makes successive improvements. For example, our model initially used a dense *adjacency matrix* representation for *G*, but subsequent optimizations led us to use a sparse *parent set* representation instead. Similarly, our MCMC method started with a naïve ‘add or remove edge’ proposal distribution; we arrived at our sparse proposal distribution (Section 2.2) after multiple refinements. Other PPLs do not allow this level of control.

### 2.4 Simulation study evaluation

We use a simulation study to answer important questions about SSPS: how does its computational expense grow with problem size? Is it able to correctly identify true edges? What is its sensitivity to errors in the prior knowledge? Simulations allow us to answer these questions in a controlled setting where we have access to ground truth.

#### 2.4.1 Data simulation process

We generate each simulated dataset as follows:

1. Sample a random adjacency matrix $A\in {\left\{0,1\right\}}^{\left|V|\times |V\right|}$, where each entry is the outcome of a Bernoulli(p) trial. *A* specifies the *structure* of a DBN. We choose p=5/|V| so that each vertex has an average of five parents. This approximates the sparsity we might see in signaling pathways. We denote the size of the original edge set as $\left|E_{0}\right|$.
2. Let the weights *β* for this DBN be drawn from a normal distribution $N(0,1/\sqrt{\left|V\right|})$. We noticed empirically that the $1/\sqrt{\left|V\right|}$ scale prevented the simulated time series from diverging to infinity.
3. Use the DBN defined by A,β to simulate *M* time courses of length *T*. We imitate the real datasets in Section 2.5 by generating M=4 time courses, each of length T=8.
4. Corrupt the adjacency matrix *A* in two steps: (i) remove $r\cdot |E_{0}|$ of the edges from *A* and (ii) add $a\cdot |E_{0}|$ spurious edges to the adjacency matrix. This corrupted graph simulates the *imperfect prior knowledge* encountered in reality. The parameters *r* and *a* control the ‘false negatives’ and ‘false positives’ in the prior knowledge, respectively.

We use a range of values for parameters |V|,r and *a*, yielding a grid of simulations summarized in Table 1. See Appendix D and Supplementary Figure S1 for additional details.

**Table 1.** Parameters that define the grid of simulated datasets in our simulation study

Parameter	Meaning	Values
|V|	Number of variables	40, 100, 200
*T*	Time course length	8
*M*	Number of time courses	4
*r*	Fraction of original edges removed	0.1, 0.5, 0.75, 1.0
*a*	Fraction of spurious edges added	0.1, 0.5, 0.75, 1.0

*Note*: There are 3×4×4=48 distinct grid points. For each one, we generate K=5 replicates for a total of 240 simulated datasets. The graph corruption parameters, *r* and *a*, range from very little error (0.1) to total corruption (1.0).

#### 2.4.2 Performance metrics

We are primarily interested in SSPS’s ability to correctly recover the structure of the underlying signaling pathway. The simulation study allows us to measure this in a setting where we have access to ground truth. We treat this as a probabilistic binary classification task, where the method assigns an *existence confidence* to each possible edge. We measure classification performance using area under the precision-recall curve (AUCPR). We use *average precision* to estimate AUCPR, as opposed to the trapezoidal rule [which tends to be overly-optimistic, see Davis and Goadrich (2006) and Flach and Kull (2015)].

Our decision to use AUCPR is motivated by the sparseness of the graphs. For sparse graphs the number of edges grows linearly with |V| while the number of possible edges grows quadratically. Hence, as |V| grows, the proportion of positive instances decreases and the classification task increasingly becomes a ‘needle-in-haystack’ scenario.

Performance measurements on simulated data come with many caveats. It is most instructive to think of simulated performance as a sanity check. Since our data simulator closely follows our modeling assumptions, poor performance would suggest serious shortcomings in our method.

### 2.5 HPN-DREAM network inference challenge evaluation

We measure SSPS’s performance on experimental data by following the evaluation outlined by the HPN-DREAM Breast Cancer Network Inference Challenge (Hill *et al.*, 2016). Signaling pathways differ across contexts—e.g. cell type and environmental conditions. The challenge is to infer these context-specific signaling pathways from time course data.

#### 2.5.1 Dataset

The HPN-DREAM challenge provides phosphorylation time course data from 32 biological contexts. These contexts arise from exposing four cell lines (BT20, BT549, MCF7 and UACC812) to eight stimuli. For each context, there are approximately M=4 time courses, each about T=7 time points in length. Cell lines have differing numbers of phosphosite measurements (i.e. differing |V|), ranging from 39 (MCF7) to 46 (BT20).

#### 2.5.2 Prior knowledge

Participants in the original challenge were free to extract prior knowledge from any existing data sources. As part of their analysis, the challenge organizers combined participants’ prior graphs into a set of edge probabilities. These *aggregate priors* summarize the participants’ collective knowledge. They were not available to participants in the original challenge, but we use them in our analyses of HPN-DREAM data. We provide them to each of the baseline methods (see Section 2.6), so the resulting performance comparisons are fair. We do not compare any of our scores to those listed by Hill *et al.* (2016) in the original challenge results.

#### 2.5.3 Performance metrics

The HPN-DREAM challenge aims to score methods by their ability to capture causal relationships between pairs of variables. It estimates this by comparing predicted *descendant sets* against experimentally generated descendant sets. More specifically, the challenge organizers exposed cells to AZD8055, an mTOR inhibitor, and observed the effects on other phosphosites. From this they determined a set of phosphosites *downstream* of mTOR in the signaling pathway. These include direct substrates of the mTOR kinase as well as indirect targets.

Comparing predicted descendants of mTOR against experimentally generated descendants of mTOR gives us a notion of *false positives* and *false negatives*. As we vary a threshold on edge probabilities, the predicted mTOR descendants change, which allows us to make a receiver operating characteristic (ROC) curve. We calculate the resulting area under the ROC curve (AUCROC) with the trapezoidal rule to score methods’ performance on the HPN-DREAM challenge. Hill *et al.* (2016) provide more details for this descendant set AUCROC scoring metric. AUCROC is sensible for this setting since each descendant set contains a large fraction of the variables. Sparsity is not an issue.

Because SSPS is stochastic we run it K=5 times per context, yielding five AUCROC scores per context. Meanwhile the baseline methods are all deterministic, requiring only one execution per context. We use a simple terminology to compare SSPS’s scores against those of other methods. In a given context, we say SSPS *dominates* another method if its *minimum* score exceeds that of the other method. Conversely, we say the other method dominates SSPS if its score exceeds SSPS’s *maximum* score. This *dominance* comparison has flaws—e.g. its results depend on the sample size *K*. However, it errs on the side of strictness and suffices as an aid for summarizing the HPN-DREAM evaluation results.

### 2.6 Baseline pathway inference algorithms

Our evaluations compare SSPS against a diverse set of baseline methods.

#### 2.6.1 Exact DBN (Hill *et al.*, 2012)

This method was an early inspiration for SSPS and is most similar to SSPS. However, the exact DBN method encounters unique practical issues when we run it on real or simulated data. The method’s computational expense increases rapidly with problem size |V| and becomes intractable unless the ‘max-indegree’ parameter is set to a small value. For example, we found that the method used more than 32 GB of RAM on problems of size |V|=100, unless max-indegree was set ≤3. Furthermore, the exact DBN method only admits prior knowledge in the form of Boolean *reference edges*, rather than continuous-valued edge confidences. We overcame this by using a threshold to map edge confidences to 1 or 0. We chose a threshold of 0.25 for the HPN-DREAM challenge evaluation because it yielded a reasonable number of prior edges. We ran Hill *et al.*’s implementation using MATLAB 2018a.

#### 2.6.2 FunChisq (Zhang and Song, 2013)

This method is based on the notion that two variables *X*, *Y* have a causal relationship if there exists a *functional dependence* Y=f(X) between them. It detects these dependencies using a χ^2^ test against the ‘no functional dependence’ null hypothesis. FunChisq was a strong competitor in the HPN-DREAM challenge, despite the fact that it uses no prior knowledge. In order to use FunChisq, one must first discretize their time course data. We followed Zhang and Song’s recommendation to use 1D *k*-means clustering for discretization. Detailed instructions are given in the HPN-DREAM challenge Supplementary Materials (Hill *et al.*, 2016). We used the FunChisq (v2.4.9.1) and Ckmeans.1d.dp (v4.3.0) R packages.

#### 2.6.3 LASSO

We included a variant of LASSO regression as a simple baseline. It incorporates prior knowledge into the typical primal formulation:

$$\hat{\beta_{j}}={\text{argmin}}_{\beta }\left\{\left||X_{+,j}-B_{j}\beta |{|}_{2}^{2}+\alpha \sum_{i=1}^{V}e^{-c_{ij}}|\beta_{i}\right|\right\},$$

where *c_ij_* is the prior confidence (either Boolean or real-valued) for edge (*i*, *j*). That is, the method uses *penalty factors* $e^{-c_{ij}}$ to discourage edges with low prior confidence. The method selects LASSO parameters, *α*, using the Bayesian Information Criterion described by Zou et al. (2007). We use GLMNet (Friedman *et al.*, 2010) via the GLMNet.jl Julia wrapper (v0.4.2).

#### 2.6.4 Prior knowledge baseline

Our most straightforward baseline simply reports the prior edge probabilities, performing no inference at all. Ideally, a Bayesian method should do no worse than the prior—new time course data should only *improve* our knowledge of the true graph. In reality, this improvement is subject to caveats about data quality and model fit.

### 2.7 SSPS software availability

We provide the SSPS code, distributed under a MIT license, via GitHub (https://github.com/gitter-lab/ssps) and archive it on Zenodo (https://doi.org/10.5281/zenodo.3939287). It includes a Snakemake workflow (Koster and Rahmann, 2012) for our full evaluation pipeline, enabling the reader to reproduce our results. The code used in this manuscript corresponds to SSPS v0.1.1.

## 3 Results

We describe evaluation results from the simulation study and HPN-DREAM network inference challenge. SSPS competes well against the baselines, with superior scalability to other DBN-based approaches.

### 3.1 Simulation study results

We compare our method to the baselines listed in Section 2.6. We focus especially on the exact DBN method of Hill *et al.* (2012), as SSPS shares many modeling assumptions with it.

#### 3.1.1 Computational expense

Because SSPS uses MCMC, the user may allow it to run for an arbitrary amount of time. With this in mind, we summarize SSPS’s timing with two numbers: (i) N/cpu-hr, the number of MCMC samples per CPU-hour and (ii) $N_{\text{eff}}/\text{cpu}-\text{hr}$, the *effective* number of samples per CPU-hour. We also measure the memory footprint per Markov chain, subject to our termination conditions. We measured these numbers for each simulation in our grid (see Table 1).

Table 2 reports average values of $N/\text{cpu}-\text{hr},\;N_{\text{eff}}/\text{cpu}-\text{hr}$, and memory footprint for each problem size. As we expect, N/cpu-hr and $N_{\text{eff}}/\text{cpu}-\text{hr}$ both decrease approximately with the inverse of |V|. In contrast, the non-monotonic memory usage requires more explanation. It results from two causes: (i) our termination condition and (ii) the sparse data structures we use to store samples. On small problems (|V|=40), the Markov chain terminates at a length of 100 000—well within the 12 h limit. On larger problems (|V|=100 or 200), the Markov chain terminates at the 12 h timeout. This accounts for the 500 MB gap between small and large problems. The *decrease* in memory usage between |V|=100 and 200 results from our sparse representations for samples. Roughly speaking, the sparse format only stores *changes* in the variables. So the memory consumption of a Markov chain depends not only on |V|, but also on the *acceptance rate* of the Metropolis–Hastings proposals. The acceptance rate is smaller for |V|=200, yielding a net decrease in memory usage.

**Table 2.** Computational expense of SSPS as a function of problem size |V|

|V|	N/cpu-hr	$N_{\text{eff}}/\text{cpu}-\text{hr}$	MB per chain
40	70 000	400	500
100	9000	140	1200
200	3000	60	1000

*Note: N* is the number of iterations completed by a Markov chain. $N_{\text{eff}}$ accounts for burnin and autocorrelation in the Markov chains, giving a more accurate sense of the method’s progress. The last column gives the approximate memory footprint of each chain. The non-monotonic memory usage is an artifact of the chain termination conditions (N>100 000 or time >12 h).

Recall that SSPS differs from more traditional MCMC approaches by nature of its parent set proposal distribution, which is specially designed for sparse graphs (see Section 2.2). When we modify SSPS to instead use a naïve uniform graph proposal, we see a striking difference in sampling efficiency. The uniform graph proposal distribution attains $N_{\text{eff}}/\text{cpu}-\text{hr}$ of 100, 10 and 0.2 for |V|=40,100 and 200, respectively—drastically smaller than those listed in Table 2 for the parent set proposal. It is possible that the traditional proposal could achieve higher $N_{\text{eff}}/\text{cpu}-\text{hr}$ by simply running faster. However, the more important consideration is how $N_{\text{eff}}/\text{cpu}-\text{hr}$ changes with |V|. Our parent set proposal distribution’s $N_{\text{eff}}/\text{cpu}-\text{hr}$ decays approximately like O(1/|V|). This is better than what we might expect from a simple analysis (Appendix B). Meanwhile, the traditional proposal distribution’s $N_{\text{eff}}/\text{cpu}-\text{hr}$ decays faster than $O(1/|V{|}^{4})$. This gap between O(1/|V|) and $O(1/|V{|}^{4})$ sampling efficiencies makes an enormous difference on large problems.

Table 3 summarizes the computational expense of the exact DBN method (Hill *et al.*, 2012). The method quickly becomes impractical as the problem size grows, unless we enforce increasingly strict in-degree restrictions. In particular, the exact DBN method’s memory cost grows exponentially with its ‘max in-degree’ parameter. The growth becomes increasingly sharp with problem size. When |V|=200, increasing the maximum in-degree from 2 to 3 makes the difference between terminating in <1 min and exceeding 32 GB of memory. Such low bounds on in-degree are unrealistic, and will likely result in poor inference quality. In comparison, SSPS makes no constraints on in-degree, and its memory usage scales well with problem size.

**Table 3.** Computational expense of the exact DBN method of Hill *et al.* (2012) measured in CPU-seconds, as a function of problem size |V| and various parameter settings

|V|	max indeg	‘linear’	‘full’
40	4	66 s	210 s
	5	770 s	3900 s
	6	6700 s	**TIMEOUT**
	7	**OOM**	**OOM**
100	3	250 s	520 s
	4	**OOM**	**OOM**
200	2	53 s	140 s
	3	**OOM**	**OOM**

*Note*: The method imposes an in-degree constraint on each vertex, shown in the ‘max indeg’ column. The columns ‘linear’ and ‘full’ correspond to different *regression modes*, i.e. which interaction terms are included in the DBN’s conditional probability distributions. Out Of Memory (‘**OOM**’) indicates that the method exceeded a 32 GB memory limit. ‘**TIMEOUT**’ indicates that the method failed to complete within 12 h.

The other baseline methods—FunChisq and LASSO—are much less computationally expensive. Both finish in seconds and require < 100 MB of memory for each simulated task. This highlights the computationally intense nature of Bayesian approaches. Not every scenario calls for Bayesian inference. However, Bayesian inference is valuable in scientific settings where we are concerned with uncertainty quantification.

#### 3.1.2 Predictive performance

The simulation study provides a setting where we have access to ‘ground truth’—the true simulated graph. We use AUCPR to score each method’s ability to recover the true graph’s edges.

**Fig. 3.**
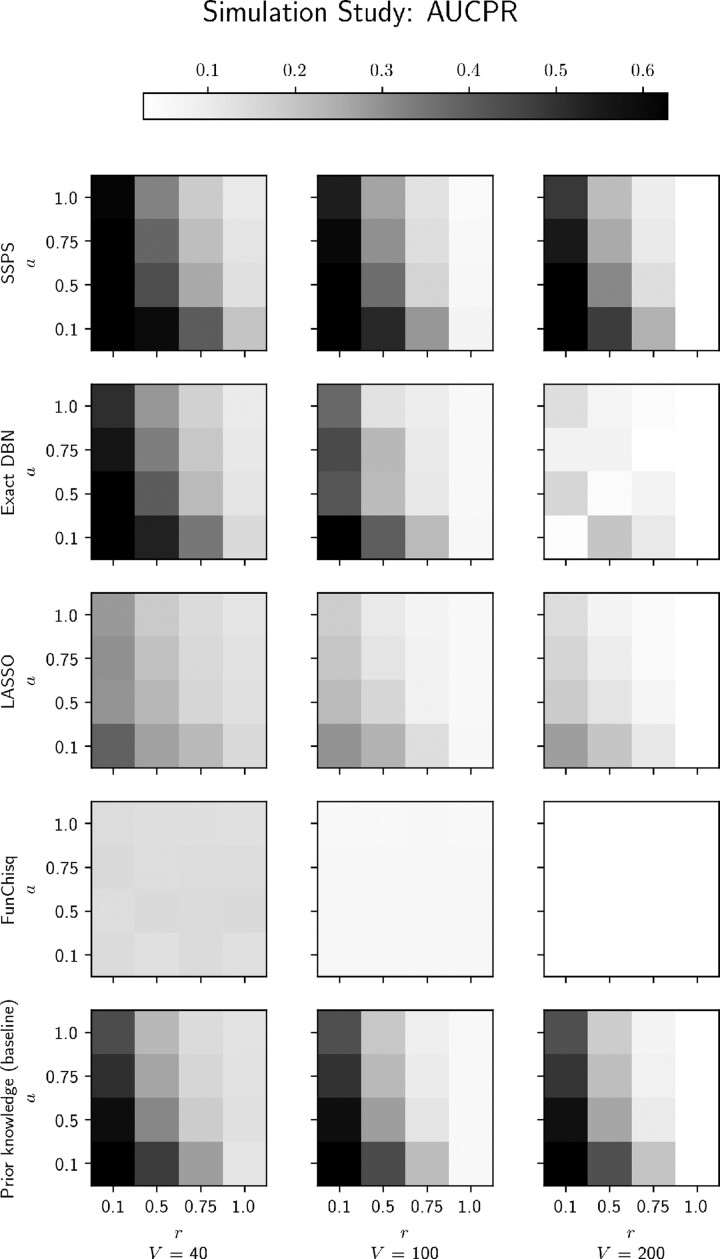
Heatmap of AUCPR values from the simulation study. Both DBN-based techniques (SSPS and the exact method) score well on this, since the data are generated by a DBN. On large problems the exact DBN method needs strict in-degree constraints, leading to poor prediction quality. LASSO and FunChisq both perform relatively weakly. See Supplementary Figure S2 for representative ROC and precision-recall curves

Figure 3 shows the AUCPR scores for our grid of simulations. Each heat map shows AUCPR as a function of graph corruption parameters, *r* and *a*. The heat maps are arranged by method and problem size |V|. Each AUCPR value is an average over five replicates. SSPS maintains fairly consistent performance across problem sizes. In contrast, the other methods’ scores decrease with problem size. For the exact DBN method, this is partially due to the small in-degree constraints imposed on the large problems. It is forced to trade model accuracy for tractability.

**Fig. 4.**
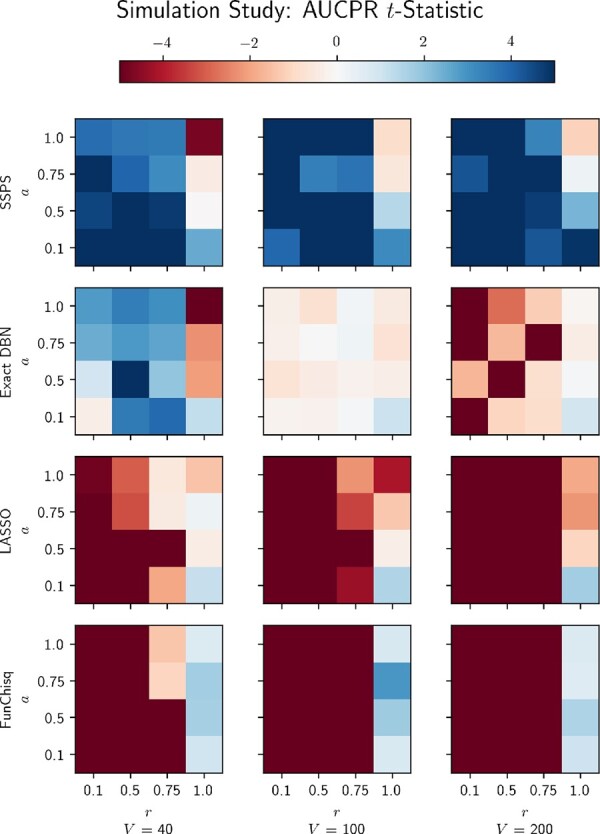
Heatmap of differential performance against the prior knowledge, measured by AUCPR paired *t*-statistics. SSPS consistently outperforms the prior knowledge across problem sizes and shows robustness to errors in the prior knowledge

Figure 4 reveals further insights into these results. It plots *differential* performance with respect to the prior knowledge, in a layout analogous to Figure 3. Specifically, it plots the *t*-statistic of each method’s AUCPR, paired with the prior baseline’s AUCPR. Whenever the prior graph has some informative edges, SSPS outperforms the prior. On the other hand, SSPS’s performance deteriorates whenever the prior contains *no* true edges (i.e. r=1). Under those circumstances FunChisq may be a better choice. Since it does not rely on prior knowledge at all, it outperforms the other methods when the prior is totally corrupted. However, we expect that in most realistic settings there exists partially-accurate prior knowledge, in which case we expect FunChisq to perform worse than SSPS.

These results confirm SSPS’s ability to identify the true network, given partially-accurate prior knowledge and time series data consistent with the modeling assumptions. SSPS is fairly robust with respect to the prior’s quality and has consistent performance across different problem sizes.

### 3.2 HPN-DREAM challenge results

**Fig. 5.**
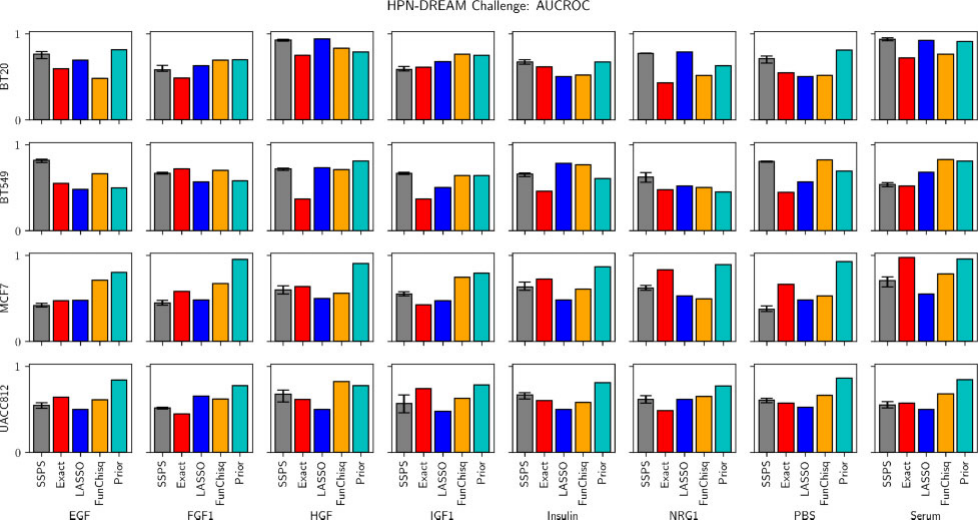
Methods’ performances across contexts in the HPN-DREAM Challenge. MCMC is stochastic, so we run SSPS 5 times; the error bars show the range of AUCROC scores. The other methods are all deterministic and require no error bars. See Supplementary Figure S3 for example predicted networks, Supplementary Figure S4 for AUCPR scores and Supplementary Figure S5 for representative ROC and precision-recall curves

We evaluated SSPS on experimental data from the HPN-DREAM challenge. The challenge includes time series phosphorylation data from 32 biological contexts: 8 stimuli applied to 4 breast cancer cell lines. Methods are scored on their ability to correctly identify the experimentally derived descendants of mTOR. Figure 5 shows bar charts comparing the methods’ AUCROC scores in each context. Appendix E provides additional details.

SSPS performs satisfactorily on this task overall. Employing terminology from Section 2.5, SSPS dominates the exact DBN method in 18 of the 32 contexts, whereas the exact DBN method dominates SSPS in only 9 contexts. Meanwhile, SSPS dominates FunChisq in 11 contexts and is dominated by FunChisq in 15. This is not surprising because FunChisq was among the top competitors in the original challenge. LASSO, on the other hand, performs poorly. SSPS dominates LASSO in 18 contexts and is dominated in only 6.

More puzzling is the strong performance of the prior knowledge baseline. SSPS dominates the aggregate prior in only 9 contexts and is dominated in 21. This is not isolated to our method. FunChisq outperforms and is outperformed by the prior knowledge in 11 and 21 contexts, respectively. The aggregate prior’s strong performance is consistent with the results from the original HPN-DREAM challenge; this prior outperformed all individual challenge submissions (Hill *et al.*, 2016). Even though the aggregate prior gives identical predictions for each context and totally ignores the time course data, it still attains better performance than the other methods. This suggests either (i) the data are relatively uninformative or (ii) the evaluation metric based on mTOR’s descendants is not sufficiently precise to measure context-specific performance. We suspect the latter, because FunChisq uses no prior knowledge but was the top performer in the HPN-DREAM challenge’s *in silico* tasks.

## 4 Discussion

We presented SSPS, a signaling pathway reconstruction technique based on DBN structure estimation. It uses MCMC to estimate the posterior probabilities of directed edges, employing a parent set proposal distribution specially designed for sparse graphs. SSPS is a Bayesian approach. It takes advantage of prior knowledge with edge-specific confidence scores and can provide uncertainty estimates on the predicted pathway relationships, which are valuable for prioritizing experimental validation.

SSPS scales to large problems more efficiently than past DBN-based techniques. On simulated data, SSPS yields superior edge predictions with robustness to flaws in the prior knowledge. Our HPN-DREAM evaluation shows SSPS performs comparably to established techniques on a community standard task. It is difficult to make stronger statements in the HPN-DREAM setting because the prior knowledge baseline performs so well and we can only evaluate the predicted mTOR descendants, not the entire pathway. However, SSPS’s scalability among Bayesian methods, strong results in the simulation, and competitive performance in the HPN-DREAM challenge make it an attractive option for further investigation of real phosphorylation datasets.

There are several potential limitations of SSPS relative to alternative pathway signaling models. Prior knowledge is not available in some organisms or biological conditions, reducing one advantage of our Bayesian approach. Although SSPS is more scalable than related DBN techniques, it would struggle to scale to proteome-wide phosphoproteomic data measuring thousands of phosphosites. For large datasets, we recommend running SSPS on a pruned version that includes only the highest intensity or most variable phosphosites. SSPS, like most DBN techniques, models only observed variables. It will erroneously exclude important pathway members, such as scaffold proteins, that are not phosphorylated. Latent variable models or background network-based algorithms are better-suited for including unphosphorylated proteins in the pathway. Background network methods can also impose global constraints on the predicted pathway structure, such as controlling the number of connected components or proteins’ reachability from relevant receptors (Köksal *et al.*, 2018).

There are many possible ways to improve SSPS. For example, it could be extended to jointly model related pathways in a hierarchical fashion, similar to Oates *et al.* (2014) and Hill *et al.* (2017). Alternatively, SSPS could be modified to accommodate causal assumptions via Pearl’s intervention operators; see the model of Spencer *et al.* (2015) for a relevant example. Combining temporal and interventional data (Cardner *et al.*, 2019) is another rich area for future work. On the algorithmic side, we could improve our MCMC procedure by adaptively tuning the parameters of its proposal distributions, as described by Gelman *et al.* (2014). Because SSPS is a probabilistic program, it is naturally extensible.

## Supplementary Material

btaa861_Supplementary_Data
