# Supplementary material for: Inferring signaling pathways with probabilistic programming
Source: Bioinformatics. 2020 Dec 29;36(Suppl 2):i822–30. doi: 10.1093/bioinformatics/btaa861 (PMC7773483; doi:10.1093/bioinformatics/btaa861)
Supplement: btaa861_Supplementary_Data [file btaa861_supplementary_data.pdf]

Supplementary Information: Inferring Signaling Pathways with Probabilistic Programming

David Merrell and Anthony Gitter

Appendix

A Model formulation details

We provide additional information about our graph prior and marginal likelihood function. We also describe some implications of SSPS's model assumptions.

Derivation of graph prior (Equation 4). We step through a more detailed derivation of SSPS's new graph prior. We begin with the original graph prior (Equation 1) and rewrite it in terms of the edge existence variables Z:

$$P(G|G',\lambda) \propto \exp\left(-\lambda|E(G)\setminus E(G')|\right)$$

$$= \exp\left(-\lambda \sum_{(i,j)\notin E(G')} z_{ij}\right) \tag{S1}$$

$$= \prod_{(i,j)\notin E(G')} e^{-\lambda z_{ij}}$$

$$= \prod_{(i,j)\notin E(G')} \left(e^{-\lambda}\right)^{z_{ij}}$$

$$\propto \left(\frac{1}{1+e^{-\lambda}}\right)^{V^2-|E(G')|} \cdot \prod_{(i,j)\notin E(G')} \left(e^{-\lambda}\right)^{z_{ij}}$$

$$= \prod_{(i,j)\notin E(G')} \left(\frac{1}{1+e^{-\lambda}}\right)\left(e^{-\lambda}\right)^{z_{ij}}$$

$$= \prod_{(i,j)\notin E(G')} \left(\frac{1}{1+e^{-\lambda}}\right)^{1-z_{ij}}\left(\frac{e^{-\lambda}}{1+e^{-\lambda}}\right)^{z_{ij}} \tag{S2}$$

Equation S2 shows the original prior is in fact a product of independent Bernoulli variables—the edge existence variables z_{ij}. Equation S2 explicitly assigns probability to the edges *not* contained in $E(G')$. However, it also implicitly assigns uniform probability to every edge *contained* in $E(G')$. We deduce that they are Bernoulli(0.5) variables, allowing us to write the prior $P(Z \mid G', \lambda)$ in the following form:

$$\prod_{(i,j)\in E(G')} \left(\frac{1}{2}\right)^{z_{ij}}\left(\frac{1}{2}\right)^{1-z_{ij}} \prod_{(i,j)\notin E(G')} \left(\frac{e^{-\lambda}}{1+e^{-\lambda}}\right)^{z_{ij}}\left(\frac{1}{1+e^{-\lambda}}\right)^{1-z_{ij}} \tag{S3}$$

just as shown in the main text.

Now we modify the prior to use continuous-valued edge confidences c_{ij} instead of Boolean reference edges $E(G')$. Intuitively, we want to restate Equation S3 as a single product over all Z variables, rather than two separate products. Our goal is to find a function $q(c_{ij})$ such that

$$P(Z \mid C,\lambda) = \prod_{(i,j)} q(c_{ij})^{z_{ij}}(1-q(c_{ij}))^{1-z_{ij}}.$$

However, in order to remain consistent with the original prior $q(c_{ij})$ ought to be monotone-increasing and satisfy these criteria:

$$q(0) = e^{-\lambda}/(1+e^{-\lambda}) \qquad \text{and} \qquad q(1) = 1/2.$$

It turns out that choosing

$$q(c_{ij}) = \frac{e^{-\lambda}}{e^{-c_{ij}\lambda}+e^{-\lambda}}$$

satisfies these requirements. This brings us to Equation 3 of the main text.

From there, it is straightforward to replace the single shared λ variable with a set of vertex-specific Λ variables and arrive at Equation 4.

Marginal likelihood function details. Equation 2 is obtained by *(i)* using a Gaussian DBN as the likelihood function for G, *(ii)* assuming certain prior distributions for the DBN parameters, and *(iii)* integrating the DBN parameters out. Specifically, let β_j and σ_j^2 $\forall j \in \{1 \ldots |V|\}$ be the DBN's weight and noise parameters, respectively. We assume an improper prior $\sigma_j^2 \propto 1/\sigma_j^2$ for the noise and a Gaussian prior for the weights:

$$\beta_j|\sigma_j^2 \sim \mathcal{N}\left(0, T\sigma_j^2(B_j^\top B_j)^{-1}\right).$$

In other words, SSPS uses an improper joint prior $P(\beta_j, \sigma_j^2) = P(\beta_j|\sigma_j^2)P(\sigma_j^2)$ with $P(\sigma_j^2)\propto 1/\sigma_j^2$. This choice allows β_j and σ_j^2 to be marginalized, yielding Equation 2.

The power $-|\text{pa}_G(j)|/2$ in Equation 2 is correct when the DBN only uses linear terms. Recall that B_j may in general contain columns of nonlinear interactions between parent variables. When that is true, the quantity $|\text{pa}_G(j)|$ should be replaced by the *width of* B_j. We elide this detail in the main text for brevity. Our implementation uses the correct exponent.

Our implementation of the marginal likelihood function employs least recently used caching to reduce redundant computation. Code profiling shows that this yields a substantial improvement to efficiency. For additional in-depth discussion of Equation 2, we recommend the supplementary materials of Hill *et al.* (2012).

Additional insights about SSPS's model assumptions. SSPS's model has interesting properties that could lead to method improvements. For example, when we replace the shared λ variable with vertex-specific Λ variables, the model effectively becomes a set of $|V|$ independent models. The plate notation in Figure 1 makes this clear; X_- is the only shared variable, and it's fully observed. This has algorithmic implications. For example, future versions of SSPS could parallelize inference at the vertex level, allocating more resources to the parent sets that converge slowly.

In the course of deriving Equation S2, we showed that our prior is a log-linear model over edge features. Equation S1 shows this most clearly. Future versions of SSPS could use the expressiveness of log-linear densities over higher-order graph features to capture richer forms of prior knowledge.

B Parent set proposal details

A key component of SSPS is its novel *parent set proposal distribution*. We motivate its design and discuss its computational complexity in greater detail.

Parent sets instead of edges. The marginal likelihood (Equation 2) is a function of the graph G. However, it depends on G only via its *parent sets*, which are encoded in the matrices B_j. Accordingly, SSPS represents G by storing a list of parents for each vertex.

It makes sense to use a proposal distribution that operates directly on SSPS's internal parent set representation. This motivates our choice of the `add-parent`, `remove-parent`, and `swap-parent` proposals listed in Section 2.2. There is a natural correspondence between *(i)* likelihood function, *(ii)* data structure, and *(iii)* proposal distribution.

Sampling efficiency. We provide some intuition for the parent set proposal's superior sampling efficiency. Let z_{ij} be a particular edge existence variable. The estimate for z_{ij} converges quickly if MCMC

updates z_{ij} frequently. Hence, as a proxy for sampling efficiency, consider the number of times z_{ij} gets updated per unit time. We decompose this quantity into three factors:

$$\frac{z_{ij} \text{ updates}}{\text{unit time}} = \epsilon \cdot \tau \cdot \alpha$$

where

$$\epsilon = \frac{\text{graph proposals}}{\text{unit time}} \qquad \tau = \frac{z_{ij} \text{ proposals}}{\text{graph proposal}}$$

$$\alpha = z_{ij} \text{ acceptance probability}$$

In other words, ϵ is the time efficiency of the proposal distribution. The factor τ is the probability that a given proposal *touches* z_{ij}. Lastly, α is the proposal's Metropolis-Hastings acceptance probability.

For a given proposal distribution, we're interested in how these factors depend on $|V|$. For simplicity of analysis, assume the Markov chain is in a typical state where the graph is sparse: $|E(G)| = O(|V|)$.

For the parent set proposal, execution time has no dependence on $|V|$ and hence $\epsilon = O(1)$. Recall that the parent set proposal resides in an outer loop, which iterates through all $|V|$ vertices. It follows that for any particular proposal there is a $1/|V|$ chance that it acts on vertex j. After choosing vertex j, there is on average a $O(1/|V|)$ chance that the proposal affects z_{ij}. This follows from the sparsity of the graph: vertex i is typically a non-parent of j and the probability of choosing it via an `add-parent` or `swap-parent` action is $O(1/|V|)$. Hence, the parent set proposal has a probability $\tau{=}O(1/|V|^2)$ of choosing z_{ij}. Lastly, the acceptance probability α has no dependence on $|V|$ and therefore $\alpha = O(1)$. The product of these factors gives an overall sampling efficiency of $O(1/|V|^2)$ for the parent set proposal.

For the uniform graph proposal, ϵ's complexity depends on the particular implementation. For sake of generosity we assume an efficient implementation with $\epsilon = O(1)$. The proposal chooses uniformly from $O(|V|^2)$ actions: add-, remove-, or reverse-edge. The probability of choosing one that affects z_{ij} is $\tau = O(1/|V|^2)$. Recall that the marginal likelihood decreases steeply with parent set size. It follows that add-edge actions will typically have low acceptance probability. Since the graph is sparse, add-edge actions are overwhelmingly probable; the probability of *not* landing on one is $O(1/|V|^2)$. If we assume the acceptance probability is high for remove-edge and reverse-edge actions, (i.e., they are accepted whenever they're proposed), then this suggests $\alpha = O(1/|V|^2)$, averaged over many proposals. The product of these factors suggests a sampling efficiency that decays like $O(1/|V|^4)$.

This gap between $O(1/|V|^2)$ and $O(1/|V|^4)$ sampling efficiencies explains most of the difference that we saw in Section 3.1. A more detailed analysis may reveal why the parent set proposal attains sampling efficiencies closer to $O(1/|V|)$ in practice.

C Probabilistic programming languages comparison

Table S1 provides a high-level comparison between PPLs.

PPL	Host language	Primary model class	Primary inference method
`Stan`	custom language	hierarchical, cont's vars	Black-box HMC
`Edward2`	Python/ TensorFlow	"deep", cont's vars	Black-box variational
`PyMC3`	Python/Theano	"deep", cont's vars	Black-box HMC
`Pyro`	Python/PyTorch	"deep", cont's vars	Black-box variational
`Gen`	Julia	discrete and cont's vars; highly flexible	Customizable MCMC

Table S1. A coarse comparison of some noteworthy PPLs. Most PPLs aim to provide a black-box interface for inference—the user is spared the difficulty of designing an inference procedure. However, this convenience comes at the cost of language restrictions. `Gen` is an exception. It provides greater expressiveness but requires the user to implement an inference program for their model. Cont's vars: continuous variables; HMC: Hamiltonian Monte Carlo.

D Simulation study details

We give additional details about the simulation study's methodology and results.

Simulation process. The simulation process described in Section 2.4 differs from SSPS's modeling assumptions in several ways. Recall that the simulator constructs a DBN to generate time series data. This simulated DBN employs nonlinear interaction terms. The simulator assumes that the data at each timepoint is a *cubic* function of the data at the previous timestep. In contrast, all of our analyses ran SSPS with an assumption of *linear* dependencies. In other words, the data contained complexities that SSPS was unable to capture. SSPS's performance in the simulation study suggests that it has some robustness to modeling assumption mismatches.

We provide an illustration of the simulation process in Figure S1. It is interesting to notice that the simulated networks do not resemble directed acyclic graphs (DAGs) in any way. They do not have any sense of directionality. Contrast this with the biological graphs shown in Figure S3. Strictly speaking these are not DAGs, but they do have an overall direction. Some vertices are source-like, and others are sink-like. Future simulations and models could be more biologically realistic if they incorporated this kind of structure.

Simulation study results. Figure S2 gives some representative ROC and PR curves from the simulation study. On problem sizes up to $|V| = 100$, SSPS and the exact DBN method yield similar curves in both ROC and PR space—though SSPS's curves clearly dominate. On larger problems the exact DBN method's performance quickly deteriorates. Computational tractability requires the exact method to impose highly restrictive in-degree constraints. These observations are consistent with the heatmaps of Figures 3 and 4 in the main text.

E HPN-DREAM challenge details

We provide additional details for the methodology and results of the HPN-DREAM challenge evaluation.

Data preprocessing. The HPN-DREAM challenge data needed to be preprocessed before it could be used by the inference methods. The choices we made during preprocessing most likely affected the inference results.

Many of the time series contain duplicate measurements. We managed this by simply averaging the duplicates. We log-transformed the time series since they were strictly positive and some methods (SSPS and exact DBN) assume normality. This probably made little difference for `FunChisq`, which discretizes the data as part of its own preprocessing.

Predicted networks. Figure S3 visualizes networks from two biological contexts in the HPN-DREAM challenge evaluation. This gives a sense of how the different inference methods' predictions differ from each other. All of the predicted networks are fairly different, though the SSPS and exact DBN predictions are more similar to each other than they are to `FunChisq`. `FunChisq` predicts more self-edges than the other methods.

In the BT549 cell line, the experimentally detected mTOR descendants include receptor proteins that would traditionally be considered upstream of mTOR in the pathway. The experimental results are reasonable due to the influence of feedback loops in signaling pathways. However, the number and positioning of the mTOR descendants highlights the differences

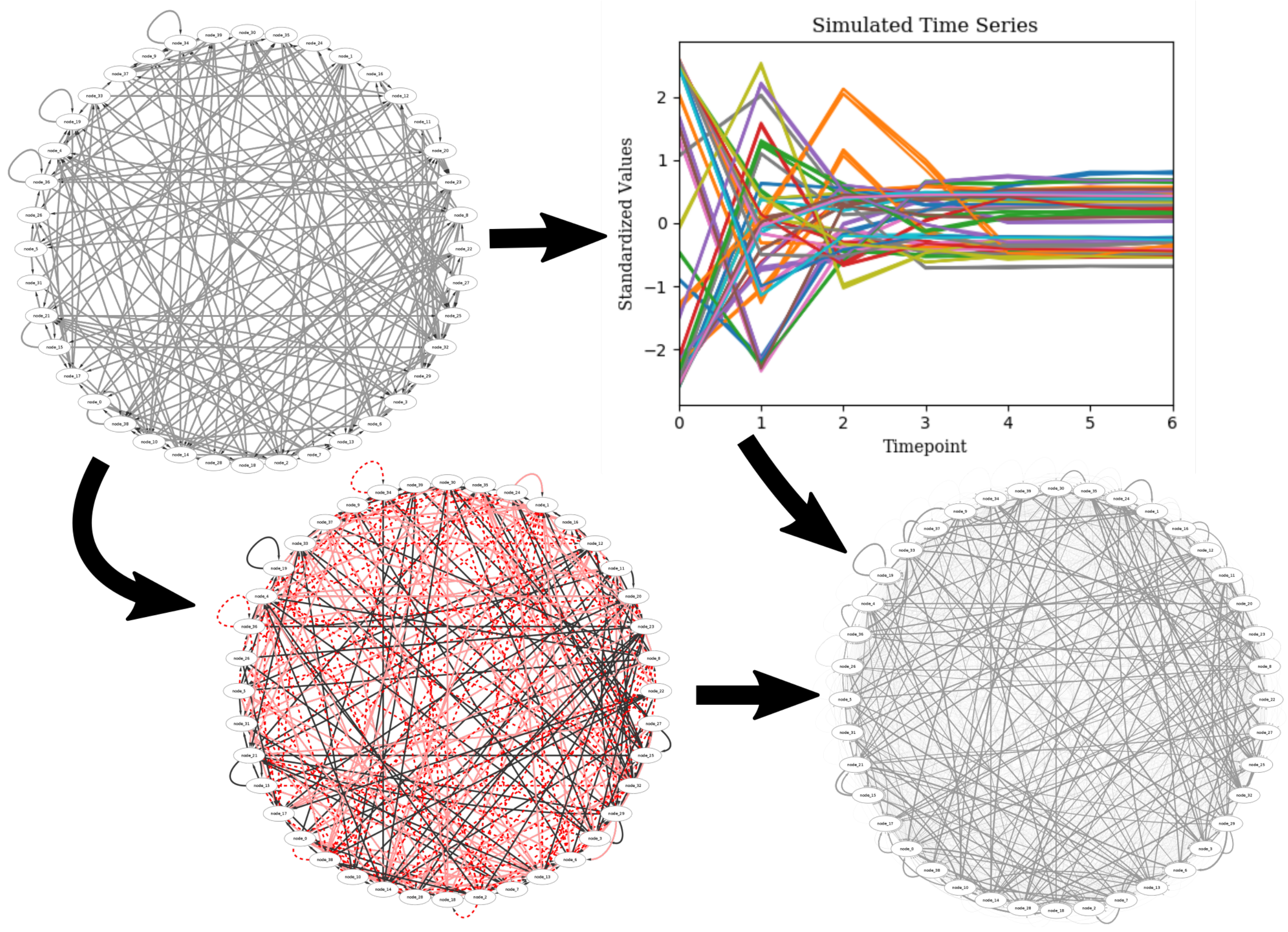

Fig. S1. A schematic of the simulation study. We randomly generate a true network (upper left) and use it to simulate a time series dataset (upper right). We corrupt the true network by adding and removing edges (lower left); solid red edges have been added, dashed red edges have been removed, and black edges are original. This corrupted network serves as partially inaccurate prior knowledge for the inference techniques. Each technique produces a predicted network (lower right) by assigning a score to each possible edge. The predicted network is evaluated with respect to the true network.

between the coarse HPN-DREAM challenge evaluation, which is based on reachability in a directed graph, and the more precise evaluation in our simulation study, where we have the edges in the ground truth network.

HPN-DREAM AUCPR. For completeness, we complement the AUCROC results of Section 3.2 with the corresponding AUCPR results. Figure S4 shows AUCPR in bar charts, with an identical layout to Figure 5.

AUCPR leads us to similar conclusions as those from AUCROC. SSPS dominates the exact DBN method in 19 contexts and is dominated in 10. Both SSPS and `FunChisq` dominate each other in 14 contexts. However, SSPS dominates the prior knowledge in only 9 contexts, and is dominated in 21. As before, we conclude that SSPS attains similar performance to established methods on this task.

ROC and PR curves. Figure S5 shows ROC and PR curves from our HPN-DREAM evaluation. We focus on two representative contexts: cell lines BT549 and MCF7, with EGF as the stimulus.

The bar charts in Figure S4 tell us that SSPS was the top performer in the (BT549, EGF) context. The ROC and PR curves are consistent with this. SSPS dominates the other methods in ROC and PR space. In contrast, SSPS was the worst performer in the (MCF7, EGF) context. The curves show SSPS performing worse than random.

The LASSO ROC and PR curves are interesting. Its ROC curves show nearly random performance. Its PR curves are straight lines. Manually inspecting its predictions yields an explanation: *(i)* LASSO gives nonzero probability to a very small number of edges; *(ii)* that small set of edges results in a very small descendant set for mTOR; *(iii)* that small descendant set is incorrect.

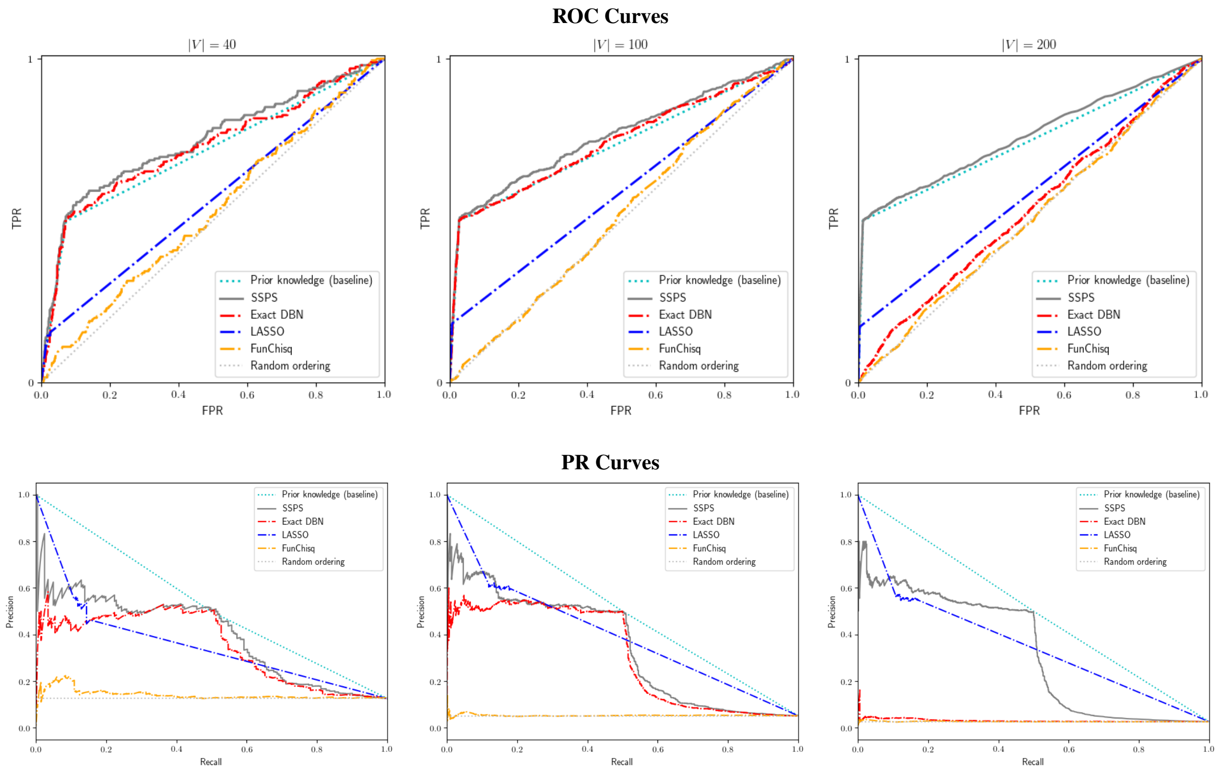

Fig. S2. Representative ROC curves (top) and PR curves (bottom) from the simulation study. We show curves for three different simulations: $|V| = 40, 100$, and 200 (left, middle, right respectively). Each of these simulations used corruption parameters $r = a = 0.5$.

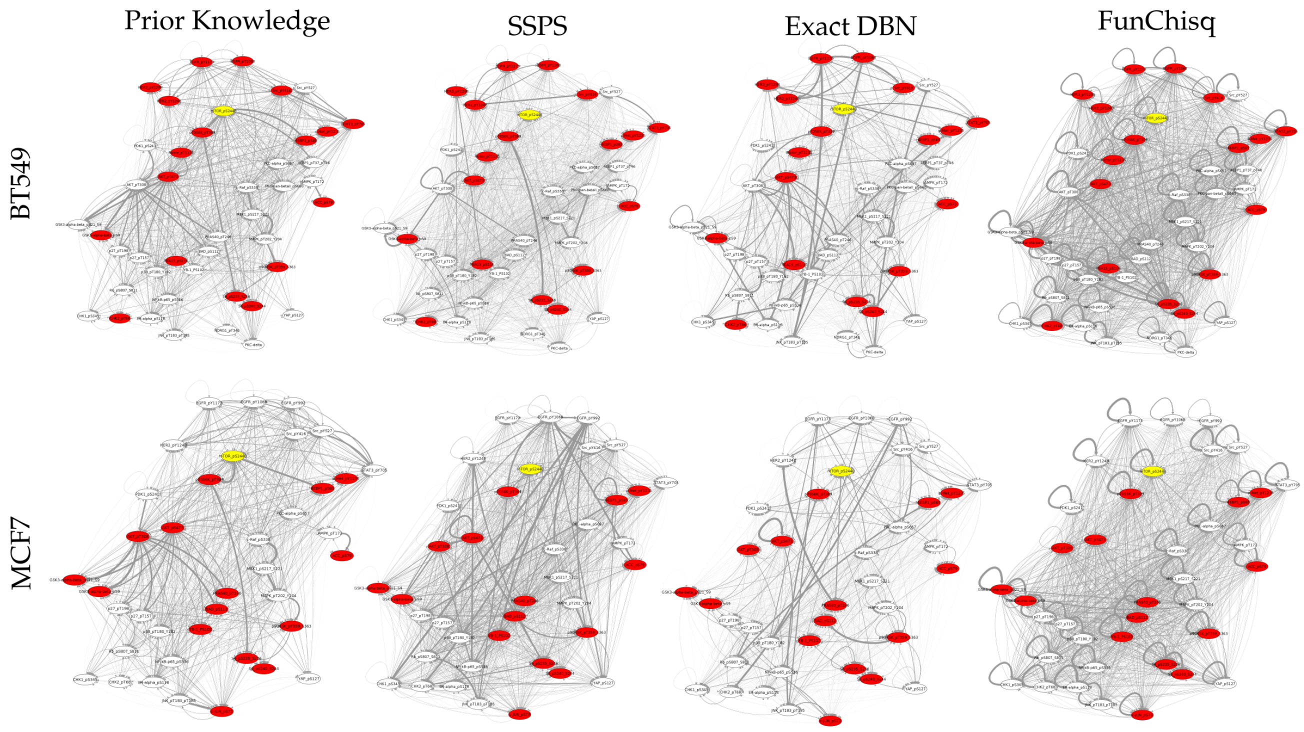

Fig. S3. Prior and predicted pathways from the HPN-DREAM challenge. We show pathways from two contexts: cell lines BT549 (top row) and MCF7 (bottom row). The stimulus is EGF for both contexts. SSPS attained the best AUCROC of all methods in the (BT549, EGF) context and the worst in the (MCF7, EGF) context. The yellow node is mTOR; red nodes are the experimentally generated ("ground truth") descendants of mTOR.

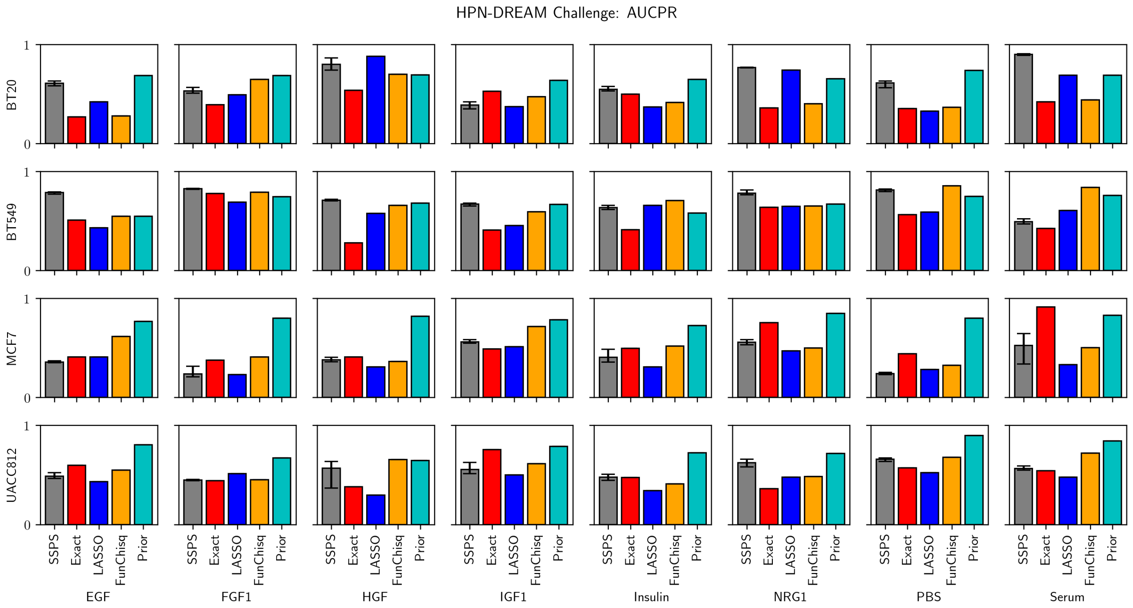

Fig. S4. A bar chart similar to Figure 5 except that it shows AUCPR rather than AUCROC. See Figure 5 for details about the layout.

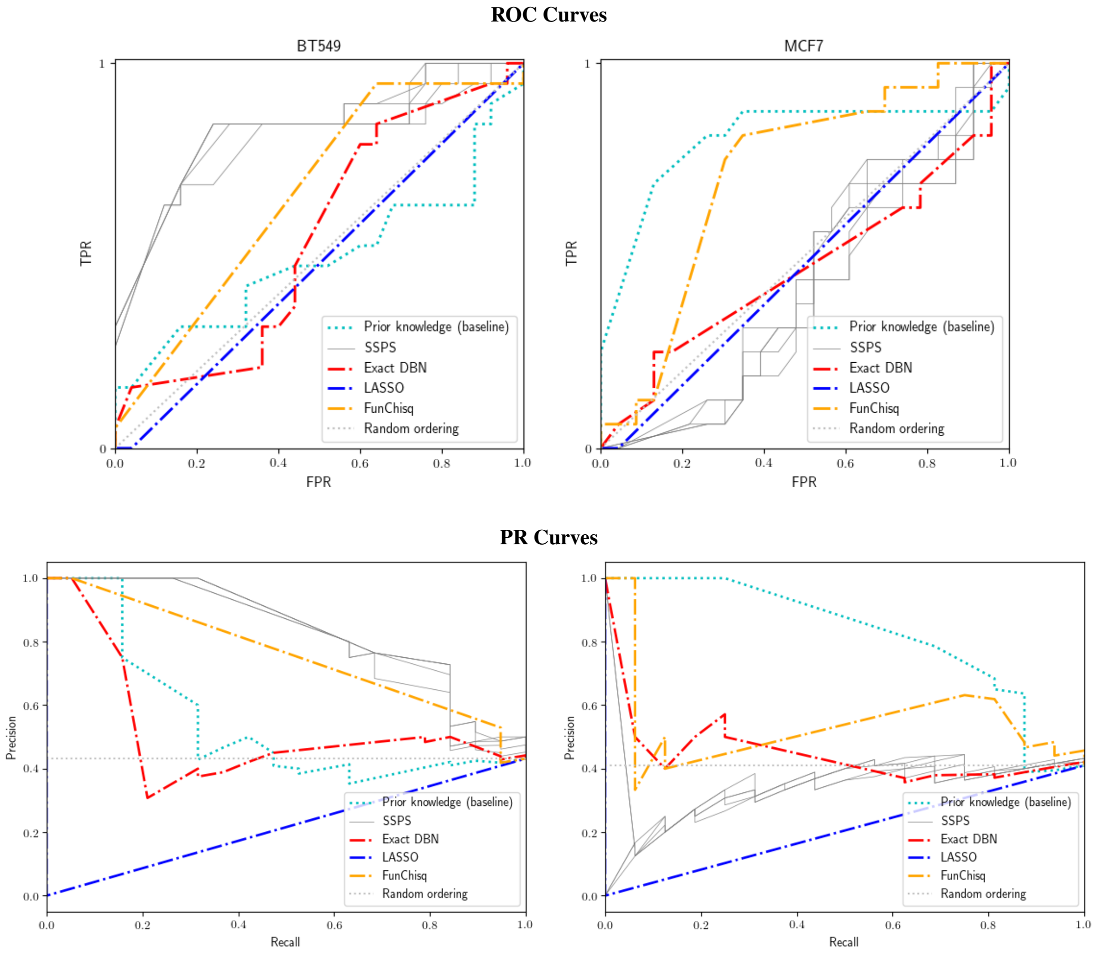

Fig. S5. ROC curves (top) and PR curves (bottom) from the HPN-DREAM challenge. We show results for two contexts: cell line BT549 (left) and MCF7 (right). The stimulus is EGF for both contexts. Since SSPS is stochastic, we show all 5 of its curves in each plot. The other methods are all deterministic, and therefore only have one curve in each plot.
